# Buoyancy effects in stagnation-point flow of Maxwell fluid utilizing non-Fourier heat flux approach

**DOI:** 10.1371/journal.pone.0192685

**Published:** 2018-05-09

**Authors:** Ammar Mushtaq, Meraj Mustafa, Tasawer Hayat, Ahmed Alsaedi

**Affiliations:** 1 Research Centre for Modeling and Simulation (RCMS), National University of Sciences and Technology (NUST), Islamabad, Pakistan; 2 School of Natural Sciences (SNS), National University of Sciences and Technology (NUST), Islamabad, Pakistan; 3 Department of Mathematics, Quaid-I-Azam University, Islamabad, Pakistan; 4 Department of Mathematics, Faculty of Science, King Abdulaziz University, Jeddah, Saudi Arabia; Vrije Universiteit Amsterdam, NETHERLANDS

## Abstract

Here we utilize a non-Fourier approach to model buoyancy aiding or opposing flow of Maxwell fluid in the region of stagnation-point towards a vertical stretchable surface. Flow field is permeated by uniform transverse magnetic field. Two different heating processes namely (i) prescribed surface temperature (PST) and (ii) constant wall temperature (CWT) are analyzed. Through suitable transformations, the similarity equations are formed which are treated numerically for a broad range of magnetic interaction parameter. The obtained solutions are compared with available articles under limiting situations and such comparisons appear convincing. The structure of boundary layer depends on a parameter measuring the ratio of free stream velocity to the stretching sheet velocity. The momentum transport via stretching boundary is opposed by both fluid relaxation time and magnetic interaction parameter. Thermal boundary layer expands as the effects of transverse magnetic field and thermal relaxation time are amplified. A reduction in heat penetration depth is anticipated for increasing values of thermal relaxation time. The variation in wall slope of temperature with increasing thermal relaxation time appears similar at any assigned value of Prandtl number. A comparative study of aiding and opposition flow situations is presented and deliberated.

## Introduction

Non-Newtonian fluids such as polymers, lubricants, granular materials, biological fluids etc. abound in daily life and in industrial processes, for example, in chemical, food processing and oil industries. The phenomenon and constitutive relations of such liquids are significantly varied and complex than the traditional viscous fluid dynamics. Viscoelastic materials display both viscous and elastic behaviors when subjected to the shearing force. The elastic effect arises due to existence of macromolecules such as polymer molecules which have a high relaxation time compared to characteristic time. Stress relaxation time is an important characteristic of these liquids which is the time required for the decay of elastic effects. Some common viscoelastic fluids are dough, yoghurt, cheese and gelled products. Viscoelastic fluid models of rate up involve one or more time derivatives and do not appear as explicit expression for stress tensor. In the past decade, Maxwell fluid model is frequently preferred by the researchers for the analysis of boundary layer problems. Maxwell fluid flow in the region of stagnation-point was analyzed by Sadeghy et al. [1] using spectral collocation point approach. They also made a comparison of results for Maxwell and second grade models. Consequences of buoyancy force on stagnation flow of Maxwell liquid near a deforming sheet were elucidated by Kumari and Nath [2]. Their results predicted that elastic effects have a retarding effect on fluid velocity. Hayat et al. [3] reported series approximations for electrically conducting flow of Maxwell fluid around a stagnation-point on a continuously deforming surface. Mukhopadhyay [4] modeled heat transfer effects in time dependent Maxwell fluid flow near a stretchable plate. Motsa et al. [5] made use of successive linearization procedure to treat the Maxwell fluid flow due to shrinking surface. A few important characteristics of boundary layer in Maxwell fluid were enlightened by Renardy and Wang [6]. Shateyi [7] provided a numerical treatment for magnetohydrodynamic (MHD) Maxwell fluid flow near a vertical surface considering the aspects of thermophoresis and chemical reaction. Bhattacharyya et al. [8] investigated multiple solutions for Maxwell fluid flow near a shrinking permeable boundary. Recently published material in this direction can be sought through refs. [9–17].

The phenomenon of heat transfer has abundant applications in numerous practical fields such as cooling towers, food processing, dispersion of temperature/moisture across groove fields, cooling of small electrical components such as microchips in computer processors, nanofluid flows, solar water cooling, enhancing performance efficiency of diesel engine oil and various others. Heat conduction model developed by Fourier [18] is of immense importance in modeling heat transfer in sundry situations. Foremost drawback of Fourier’s approach is that it gives a paradoxical prediction that any initial disturbance would instantly alter the medium under observation. To overcome this drawback, a successful generalization to Fourier heat flux theory was devised by Cattaneo [19]. He used the concept of thermal relaxation time which refers to the time needed to achieve steady-state conduction in volume element when it is subjected to temperature differences. To preserve objectivity constraint, Christov [20] used the Oldroyd’s upper-convected derivative in place of usual time derivative in Cattaneo’s model to formulate energy equation. The Cattaneo-Christov approach was utilized by Straughan [21] to inspect convection in a horizontal layer of incompressible viscous fluid. Tibullo and Zampoli [22] proved uniqueness for incompressible flow problems based on Cattaneo-Christov model. Haddad [23] explored instabilities associated with the thermal transport in Brinkman layer with thermal relaxation effects. Han et al. [24] developed series approximations for Maxwell fluid flow near a deformable surface considering the aspects of Navier slip and Cattaneo-Christov conduction. Thermal relaxation effects in rotating viscoelastic fluid flow were analyzed by Mustafa [25]. He used both numerical and analytical techniques to treat the governing non-linear system. Khan et al. [26] reported simulations for viscoelastic fluid flow induced by an exponentially deforming surface considering a non-Fourier approach. Hayat et al. [27] investigated the onset of Cattaneo-Christov conduction for swirling flow of Jeffrey fluid past a porous surface. Mushtaq et al. [28] analyzed the Sakiadis flow in the framework of Cattaneo-Christov theory using two numerical approaches. Salahuddin et al. [29] examined the behavior of Lorentz force on Williamson fluid flow due to deforming sheet with thermal relaxation effects. Recently, a number of studies featuring Cattaneo-Christov model are published (see [30–34] and refs. there in.).

Stagnation flows are particularly important in predicting drag coefficient near stagnation region of bodies in high speed flows. Fluid flow around a stagnation-point towards a stretchable sheet has been a compelling research topic because it is met in many metal working and polymer extrusion processes. The pioneering study of Heimenz [35] concerning the plane stagnation-point flow has led to may subsequent research activities. For example, Mahapatra et al. [36] modeled stagnation-point flow of conducting power-law fluid bounded by a stretchable surface. In this study, numerical calculations were made for full range of magnetic interaction parameter. Consequence of wall permeability on the stagnation-point flow near a shrinking sheet was discussed by Bhattacharyya et al. [37]. In another study, Bhattacharyya et al. [38] provided numerical analysis for MHD fluid flow around a stagnation-point with chemically reactive solute. Bhattacharyya [39] presented dual solutions for thermal transport in stagnation-point flow considering variable heat flux at the boundary. Also, Bhattacharyya [40] examined solute transfer in stagnation-point flow caused by shrinking surface with diffusive mass fluid conditions.

Inspired by the aforementioned studies, we intend to investigate the onset of mixed convection in Maxwell fluid flow due to heated or cooled vertical surface utilizing Cattaneo-Christov heat flux model. Flow field is influenced by vertical magnetic field of uniform strength. Studies presented through [41–47] demonstrate the fact that buoyancy forces resulting from the heating of the deformable surfaces are useful in terms of drag reduction and heat transfer intensification. Unlike previous studies [2], [3], [4] and [9], we consider the correct form of body force terms representing magnetic and gravitational potentials. Conventional transformations are adopted to extract local similarity equations which are treated via efficient shooting approach, the details of which can be found in [28]. For validation purpose, the results are compared with published papers in special cases and found in complete agreement. Alternative computational approaches for similar kind of boundary layer problems can be seen through [48–51] and studies there in. The impacts of important parameters on the momentum and energy transport are the main concerns of this investigation. The rest of the paper is arranged in the following manner. Mathematical formulation is covered in the next section. Section 3 gives a detailed description of the employed numerical treatment. In section 4, physical description to the behavior of emerging parameters is assigned graphically. Finally, the conclusion section highlighting major results is presented.

## Problem formulation

**Fig 1:**
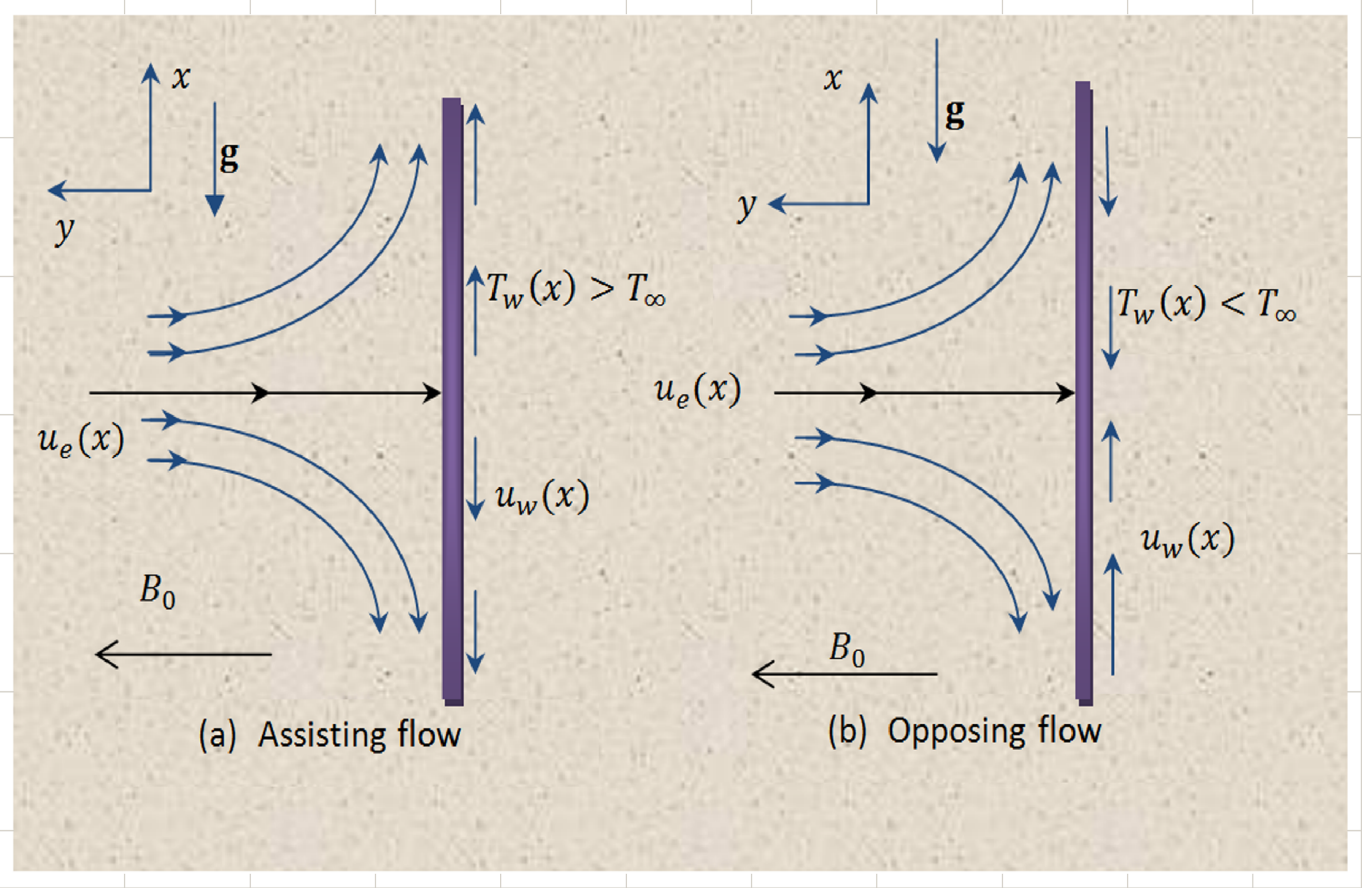
Physical model and coordinate system.

Consider a laminar viscoelastic fluid flow adjacent to a vertical elastic sheet with *u* and *v* denoting velocity components along *x*− and *y*− directions in which the coordinate *x* extends along the sheet and *y* is normal to it. Let us assume that the surface stretches in *x*− direction with velocity *u*_*w*_(*x*) = *ax* and *u*_*e*_(*x*) = *cx* denotes the velocity of external flow where *a* and *c* are positive constants. The buoyancy force resulting due to density differences aids or opposes the external flow when it is directed towards or opposite to the external stream. The conducting Maxwell fluid is exposed to transverse magnetic field of strength *B*_0_ (see Fig 1). Under low magnetic Reynolds number, induced magnetic field can be ignored in comparison with the applied magnetic field. There is no electric field. Thus if *σ* denotes the fluid electrical conductivity, the components of Lorentz force vector become (−*σB*_0_^2^*u*,−*σB*_0_^2^*v*,0). The function *T*_*w*_(*x*) = *T*_∞_ + *bx* prescribes the wall temperature in which *b* is a constant and *T*_∞_ represents the temperature of quiescent fluid. Utilizing the Oberback-Boussinesq approximation, equations representing Maxwell fluid motion with heat transfer are given below (see refs. [14] and [20]):

$$u_{x}+v_{y}=0$$ (1)

$$\begin{array}{l} uu_{x}+vu_{y}+\lambda_{1}\left(u^{2}u_{xx}+v^{2}u_{yy}+2uvu_{xy}\right)=u_{e}{\left(u_{e}\right)}_{x}+\nu u_{yy}-\frac{\sigma }{\rho }B_{0}^{2}\left(u-u_{e}+\lambda_{1}vu_{y}\right)\\ +g\beta_{T}\left[\left(T-T_{\infty }\right)+\lambda_{1}\left\{uT_{x}+vT_{y}-u_{x}\left(T-T_{\infty }\right)\right\}\right], \end{array}$$ (2)

$$\rho C_{p}\left(uT_{x}+vT_{y}\right)=-\nabla \cdot q,$$ (3)

where *λ*_1_ stands for fluid relaxation time, *ν* denotes the kinematic viscosity, *ρ* is the fluid density, *β*_*T*_ stands for the coefficient of thermal expansion, *C*_*p*_ denotes the specific heat capacity and **q** the heat flux vector. Using Cattaneo-Christov theory, the heat flux **q** obeys the following relation [20, 24]:

$$q+\lambda_{2}\left(q_{t}+V\cdot \nabla q-q\cdot \nabla V+(\nabla \cdot V)q\right)=-k\nabla T,$$ (4)

in which *k* denotes the fluid thermal conductivity and *λ*_2_ stands for thermal relaxation time. Taking divergence of Eq (4) and then utilizing Eq (3), one arrives at the following equation:

$$uT_{x}+vT_{y}+\lambda_{2}\left\{u^{2}T_{xx}+v^{2}T_{yy}+2uvT_{xy}+\left(uu_{x}+vu_{y}\right)T_{x}+\left(uv_{x}+vv_{y}\right)T_{y}\right\}=\alpha T_{yy}.$$ (5)

For detailed derivation of Eq (5), the readers are referred to the article by Christov [20]. The boundary conditions assume the following forms:

$$u(x,0)=u_{w}(x)=ax,\;T(x,0)=\{\begin{array}{c} T_{\infty }+bx\;(\mathrm{PST})\\ T_{w}\;(\mathrm{CWT}) \end{array},$$ (6)

v(x,0)=0, (7)

$$u\to u_{e}(x)=cx,\;v\to v_{e}(y)=-cy\;\mathrm{as}\;y\to \infty ,$$ (8)

$$T\to T_{\infty }\;\mathrm{as}\;y\to \infty .$$ (9)

Conditions given in (6) indicates no-slip at the wall, condition (7) represent impermeability at the boundary, condition (8) indicates that viscous effects vanish at far distance from the surface and condition (9) signifies no temperature variation far from the boundary.

Defining the non-dimensional horizontal distance *ζ* = *y*(*a*/*ν*)^1/2^, we seek the similarity solutions of (1), (2) and (5) of the following forms:

$$u=axF^{\prime }(\zeta ),\;v=-{\left(\nu a\right)}^{1/2}F(\zeta ),\;\theta (\zeta )=\left(T-T_{\infty }\right)/\left(T_{w}-T_{\infty }\right).$$ (10)

Eq (1) is satisfied by transformations (10), while Eqs (2), (5) and (6)–(9) convert into the following ordinary differential equations:

$$\begin{array}{l} F^{\prime \prime \prime }+\left(1+M\beta \right)FF^{\prime \prime }-{F^{\prime }}^{2}+\beta \left(2FF^{\prime }F^{\prime \prime }-F^{2}F^{\prime \prime \prime }\right)-MF^{\prime }\\ +\lambda \left(\theta -\beta F\theta^{\prime }\right)+M\frac{c}{a}+\frac{c^{2}}{a^{2}}=0, \end{array}$$ (11)

$$\frac{1}{\mathrm{Pr}}\theta^{\prime \prime }+F\theta^{\prime }-F^{\prime }\theta -\gamma \left(F^{2}\theta^{\prime \prime }+FF^{\prime \prime }\theta +{F^{\prime }}^{2}\theta -FF^{\prime }\theta^{\prime }\right)=0\;(\mathrm{PST}),$$ (12)

$$\frac{1}{\mathrm{Pr}}\theta^{\prime \prime }+F\theta^{\prime }-\gamma \left(F^{2}\theta^{\prime \prime }-FF^{\prime }\theta^{\prime }\right)=0\;(\mathrm{CWT}),$$ (13)

$$\begin{array}{l} F=0,\;F^{\prime }=1,\;\theta =1\;\mathrm{at}\;\zeta =0,\\ F^{\prime }\to \frac{c}{a},\;\theta \to 0\;\mathrm{as}\;\zeta \to \infty . \end{array}$$ (14)

In Eq (8), *λ* = *Gr*_*x*_/*Re*_*x*_^2^ is the mixed convection parameter (also called Richardson number) in which *Gr*_*x*_ = *gβ*_*T*_(*T*_*w*_ − *T*_∞_)*x*^3^/*ν*^2^ denotes the local Grashof number and Re_*x*_ = *u*_*w*_*x*/ν is the local Reynolds number. For positive values of *λ* we have *T*_*w*_ > *T*_∞_ in the upper half (where *x* > 0) while *T*_*w*_ < *T*_∞_ in the lower half (where *x* < 0). In this situation, buoyancy force acts in the same direction as that of free stream velocity in both upper and lower halves, thereby assisting the fluid flow. For negative *λ*, buoyancy force is directed opposite to the external stream in both upper and lower halves. The other parameters appearing in (11) and (12)-(13) are given below:

$$M=\sigma B_{0}{}^{2}/\rho a,\;\mathrm{Pr}=\nu /\alpha ,\;\beta =\lambda_{1}a,\;\gamma =\lambda_{2}a,$$ (15)

where *M* denotes the magnetic interaction parameter, Pr denotes the Prandtl number, *β* stands for fluid relaxation time and *γ* for thermal relaxation time. A list of all symbols is shown in Table 1. Note that Eqs (11, 12 and13) reduce to the viscous fluid case when *β* = 0. Also, the analysis for usual Fourier law can be recovered by selecting *γ* = 0.

**Table 1:** List of symbols.

*x*,*y* Cartesian coordinates (m)	*Re*_*x*_ local Reynolds number
(*u*,*v*) velocity components along *x-*and *y-*directions respectively (ms^-1^)	*Greek symbols*
(*u*_*e*_,*v*_*e*_) external flow velocity components in (ms^-1^)	*ζ* similarity variable
*u*_*w*_ velocity of stretching sheet (ms^-1^)	*σ* electrical conductivity (s.m^-1^)
*a*,*c* positive constants (s^-1^)	*ρ* fluid density (kg.m^-3^)
*B*_0_ magnetic field strength (Nm^-1^A^-1^)	*ν* kinematic viscosity (m^2^s^-1^)
*M* magnetic interaction parameter	*α* thermal diffusivity (m^2^s^-1^)
*g* gravitational acceleration (ms^-2^)	*λ* Richardson number
*T* fluid temperature (K)	*λ*_1_ fluid relaxation time (s)
*T*_*w*_ wall temperature (K)	*λ*_1_ heat flux relaxation time (s)
*T*_∞_ ambient fluid temperature (K)	*γ* dimensionless thermal relaxation time
*C*_*p*_ specific heat (Jkg^−1^K^−1^)	*β* dimensionless fluid relaxation time
**q** heat flux(Wm^-2^)	*β*_*T*_ coefficient of thermal expansion (K^−1^)
*k* thermal conductivity (Wm^-1^K^−1^)	*θ* dimensionless temperature
*F* dimensionless stream function	*Subscripts*
*Pr* Prandtl number	*w* condition at the wall
*Gr*_*x*_ local Grashof number	∞ condition at infinity

## Numerical procedure

Here we discuss the numerical treatment of governing Eqs. (11) and (12) using shooting method coupled with Runge-Kutta method of fifth-order and the Newton’s method. Making use of the substitutions *y*_1_ = *F*,*y*_2_ = *F*′,*y*_3_ = *F*″,*y*_4_ = *θ*,*y*_5_ = *θ*′, an initial value problem consisting of five first order ordinary differential equations is obtained:

$$\begin{array}{l} \begin{array}{cc} y_{1}{}^{\prime }=y_{2}; & y_{1}(0)=0\\ y_{2}{}^{\prime }=y_{3}; & y_{2}(0)=1 \end{array}\\ \begin{array}{cc} y_{3}{}^{\prime }=\frac{y_{2}{}^{2}-(1+M\beta )y_{1}y_{3}-2\beta y_{1}y_{2}y_{3}-\lambda (y_{4}-\beta y_{1}y_{5})+M(y_{2}-c/a)-{\left(c/a\right)}^{2}}{1-\beta y_{1}{}^{2}}; & y_{3}(0)=s_{1} \end{array}\\ \begin{array}{cc} y_{4}{}^{\prime }=y_{5}; & y_{4}(0)=1 \end{array}\\ \begin{array}{cc} y_{5}{}^{\prime }=\frac{\mathrm{Pr}\left\{y_{2}y_{4}-y_{1}y_{5}+\gamma (y_{1}y_{3}y_{4}+y_{2}{}^{2}y_{4}-y_{1}y_{2}y_{5})\right\}}{1-\mathrm{Pr}\gamma y_{1}{}^{2}}; & y_{5}(0)=s_{2} \end{array}. \end{array}$$ (16)

We replace infinity (∞) by a number, say *ζ*_∞_, which is initially taken as low as 2 and then proceed with the numerical integration of system (16) by assigning suitable guesses for the missing slopes *s*_1_ = *F*″(0) and *s*_2_ = *θ*′(0). It means that a solution to the system (16) will depend on similarity variable *ζ* and the missing slopes *s*_1_ and *s*_2_. Here the mesh size *h* = 0.01 is considered in numerical integration. Thus conditions for *F*′ and *θ* at infinity can be expressed as *y*_2_(*ζ*_∞_,*s*_1_,*s*_2_) − (*c*/*a*) = 0 and *y*_4_(*ζ*_∞_,*s*_1_,*s*_2_) = 0 respectively. These algebraic equations are solved for *s*_1_ and *s*_2_ by Newton’s method. Solutions for *s*_1_ and *s*_2_ are employed in (16) and the system is integrated at a higher *ζ*_∞_, say *ζ*_∞_ = 4. We repeat this process for different *ζ*_∞_(say *ζ*_∞_ = 5,6,7etc.) until the solutions for *s*_1_ and *s*_2_ become independent of *ζ*_∞_. In Newton’s method, computer code is designed to perform maximum 30 iterations. Our computations have shown that such number of iterations is sufficient to fulfill the desired tolerance of 10^−7^ in all the considered cases.

## Numerical results and discussion

We modeled the aiding or opposing mixed convection viscoelastic fluid flow adjacent to a heated vertical surface utilizing the novel Cattaneo-Christov model. A convenient shooting technique is implemented to compute the governing similarity equations. In Table 2, the values of *F*″(0) are compared with those of Mahapatra et al. [36] for broad range of magnetic interaction parameter *M* in Newtonian limit (*β* = 0). Present numerical results are in complete agreement with [36] for all reported values of *M*. For a further check, we made comparison of values of *F*″(0) with Mustafa et al. [34], Abel et al. [52] and Megahed [53] for the forced convection flow situation (*λ* = *c*/*a* = 0). Again we witness a very good agreement for all chosen values of Deborah number *β* (see Table 3). The values of wall temperature slope *θ*′(0) for various parameter values are listed in Table 4. It is indicated in [48] that Prandtl number for non-Newtonian fluids is usually large. Hence numerical calculations are made at Pr = 10 and Pr = 25. An increasing trend in |*θ*′(0)| is found for increasing values of dimensionless thermal relaxation time *γ*. CPU time for the numerical values computed in Table 4 is mentioned against each simulation.

**Table 2:** Comparison of velocity gradient |*F*″(0)| with that of Mahapatra et al. [36] when *β* = *λ* = 0.

*M*	(*c*/*a*) = 2		(*c*/*a*) = 0.2	
	[36]	Present	[36]	Present
0.0	2.0175	2.01750	0.9181	0.91811
0.5	2.1363	2.13632	1.0768	1.07682
1.0	2.2491	2.24910	1.2156	1.21562
1.5	2.3567	2.35667	1.3404	1.34038
2.0	2.4597	2.45967	1.4546	1.45460
3.0	2.6540	2.65398	1.6569	1.65979
5.0	3.0058	3.00578	2.0085	2.00847
10	3.7447	3.74472	2.6894	2.68944
20	4.9004	4.90037	3.6922	3.69223
40	6.6339	6.63381	5.1412	5.14123
60	8.0002	8.00032	6.2635	6.26356
80	9.1642	9.16537	7.2136	7.21333
100	10.1934	10.19819	8.052	8.05184
200	14.2825	14.28291	11.3491	11.35042
300	17.4127	17.43563	13.8537	13.88640
500	22.4499	22.44996	17.8617	17.91178
1000	31.6858	31.68596	25.1163	25.31466

**Table 3:** Comparison with wall velocity gradient −*F*″(0) obtained by Mustafa et al. [34], Abel et al. [52] and Megahed [53] for different values of *β* when *λ* = (*c*/*a*) = 0.

*β*	Mustafa et al. [34]	Abel et al. [52]	Megahed [53]		Present	
				*ζ*_∞_ = 2	*ζ*_∞_ = 5	*ζ*_∞_ = 10
0	1.000000	0.999962	0.999978	1.000000	1.000000	1.000000
0.2	1.051890	1.051948	1.051945	1.051921	1.051890	1.051890
0.4	1.101903	1.101850	1.101848	1.101789	1.101903	1.101903
0.6	1.150137	1.150163	1.150160	1.150168	1.150137	1.150137
0.8	1.196711	1.196692	1.196690	1.196682	1.196711	1.196711
1.2	1.285363	1.285257	1.285253	1.285324	1.285363	1.285363
1.6	1.368758	1.368641	1.368641	1.368715	1.368758	1.368758
2.0	1.447651	1.447617	1.447616	1.447639	1.447651	1.447651

**Table 4:** Computational results of −*θ*′(0) for varying values of *γ*,(*c*/*a*)and *β* with Pr = 10 and *M* = 5.

			Pr = 10		Pr = 25	
*γ*	(*c*/*a*)	*β*	Assisting Flow *λ* = 1	Opposing Flow *λ* = −1	Assisting Flow *λ* = 1	Opposing Flow *λ* = −1
0.2	0.3	0.2	3.978577 (32.270 sec)	3.910338 (35.197 sec)	6.551186 (60.005 sec)	6.50699 (63.613 sec)
0.4			4.354726 (13.898 sec)	4.284905 (23.047 sec)	7.128445 (36.352 sec)	7.086098 (59.329 sec)
0.6			4.693003 (45.715 sec)	4.613185 (57.069 sec)	7.623144 (13.964 sec)	7.555093 (13.637 sec)
0.8			4.959215 (16.371 sec)	4.932692 (27.048 sec)	7.947736 (8.273 sec)	7.915262 (8.407 sec)
0	0	0.2	3.357325 (2.481 sec)	3.270179 (2.524 sec)	5.659030 (2.871 sec)	5.609332 (2.877 sec)
	0.4		3.641242 (2.737 sec)	3.588133 (2.606 sec)	5.921715 (2.925 sec)	5.883671 (2.921 sec)
	0.6		3.761313 (2.837 sec)	3.715780 (2.651 sec)	6.044662 (2.961 sec)	6.010370 (2.971 sec)
	1.2		4.084024 (2.760 sec)	4.051657 (2.565 sec)	6.395400 (3.526 sec)	6.368817 (2.060 sec)
0.2	0.3	0	4.002944 (38.038 sec)	3.945292 (35.149 sec)	6.570579 (67.316 sec)	6.534230 (60.089 sec)
		0.2	3.978577 (32.270 sec)	3.910338 (35.197 sec)	6.551186 (60.005 sec)	6.50699 (63.613 sec)
		0.4	3.954021 (32.740 sec)	3.875656 (28.188 sec)	6.530987 (67.533 sec)	6.478746 (64.059 sec)
		0.8	3.905982 (27.809 sec)	3.808797 (23.860 sec)	6.488601 (63.526 sec)	6.420623 (61.060 sec)

**Fig 2:**
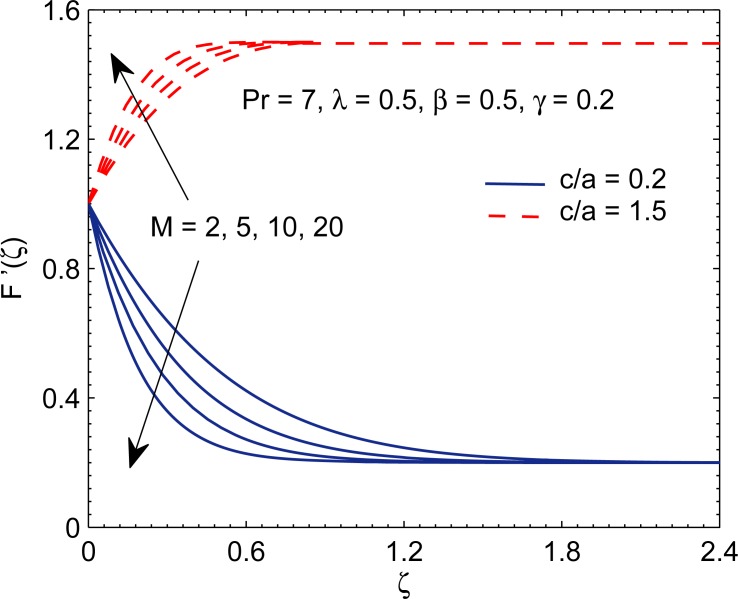
Curves of *F*′ for various values of magnetic interaction parameter *M*.

For fixed values of mixed convection parameter *λ* and Deborah number *β*, the curves of *F*′(*ζ*) representing *x*−component of velocity are portrayed in Fig 2 for different values of magnetic interaction parameter *M* and velocity ratio (*c*/*a*). Velocity parallel to the surface decreases with increasing *M* for (*c*/*a*) < 1 but increases with increases *M* for (*c*/*a*) > 1. For any value of velocity ratio (*c*/*a*) we witness a decreasing trend in boundary layer thickness for increasing values of *M*. It is the consequence of the fact that magnetic field sets up a Lorentz force in transverse direction which retards the momentum transport phenomenon.

**Fig 3:**
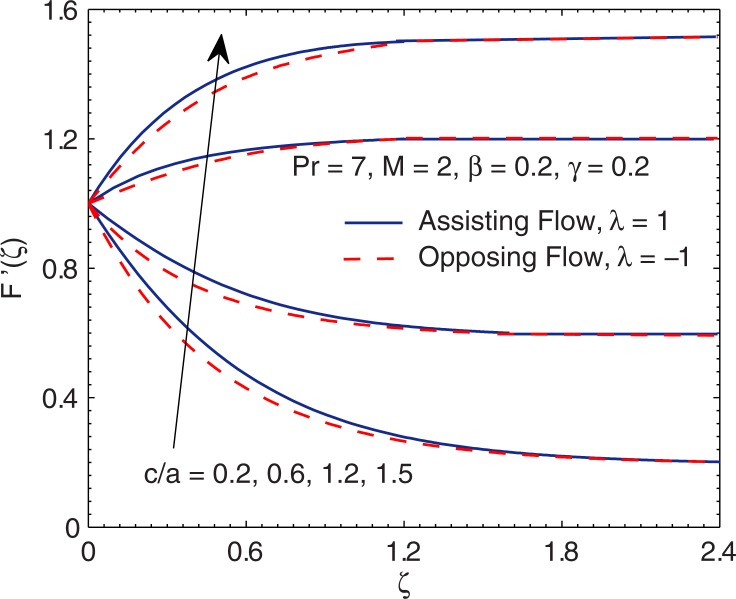
Curves of *F*′ for various values of velocity ratio parameter (*c*/*a*).

Fig 3 shows the velocity curves *F*′(*ζ*) for varying velocity ratio (*c*/*a*) in both assisting and opposing flow regimes. An increase in (c/a) can be realized by increasing free stream velocity while maintaining the same stretching rate (or by decreasing the stretching rate while keeping the free stream velocity fixed). We observe that *F*′(*ζ*) is proportional to (*c*/*a*) whereas boundary layer thickness decreases with increasing (*c*/*a*). Physically an increment in (*c*/*a*) implies a reduction in the straining motion near the surface which in turn reduces the thickness of hydrodynamic boundary layer.

**Fig 4:**
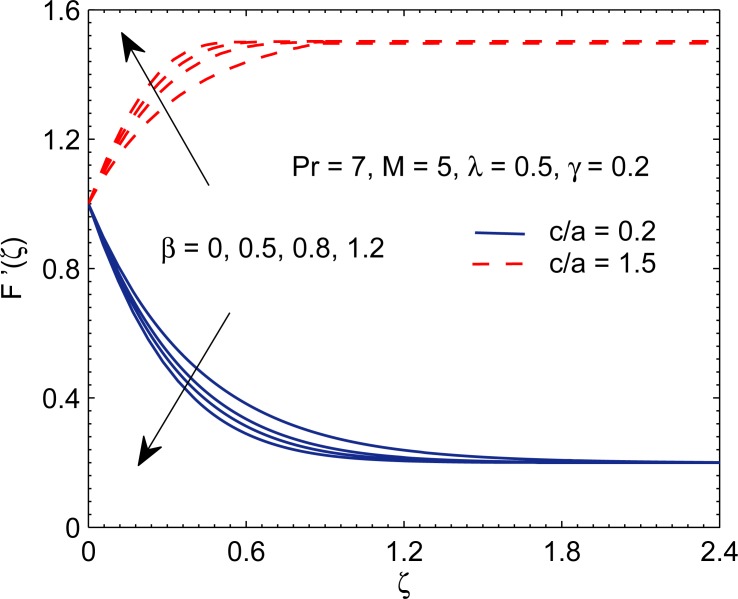
Curves of *F*′ for various values of Deborah number *β*.

In Fig 4, we present the change in velocity profile *F*′(*ζ*) with the variation in Deborah number *β*. In assisting or opposing flow regime, fluid flow in vertical direction accelerates when *β* becomes large for (*c*/*a*) > 1 while opposite effect is observed for (*c*/*a*) < 1. Notably, a decreasing trend in boundary layer thickness is found for increasing *β* and such outcome persists for any assigned value of (*c*/*a*). This is explained as follows. At low Deborah number, stress relaxation is fast in comparison to the observation time scale, and hence fluid has solid-like response to the shearing force. At higher Deborah number, the fluid response resembles closely to that of an elastic solid substance. In this situation, the boundary layer thickness does not grow as fast as for smaller Deborah number.

**Fig 5:**
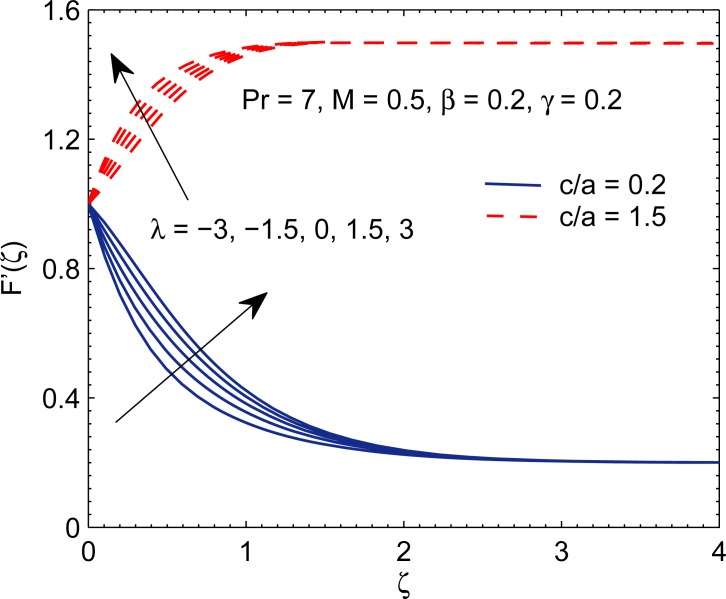
Profiles of *F*′ for different values of mixed convection parameter *γ*.

Fig 5 depicts the change in velocity curves by varying mixed convection parameter *λ*. It is observed that velocity in *x*−direction has direct relationship with *λ*. The result is in accordance with those reported by earlier studies (for instance see Kumari and Nath [9], Ali et al. [44] etc.). This trend follows from the fact that positive *λ* acts as favorable pressure gradient which accelerates the fluid flow in the boundary layer.

**Fig 6:**
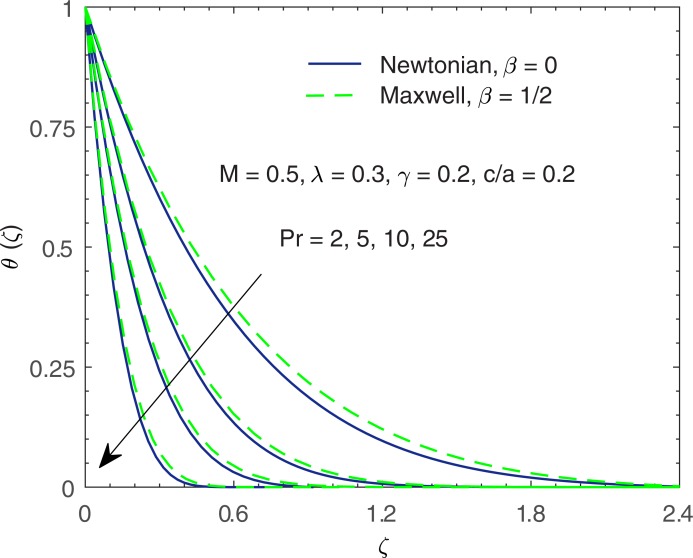
Profiles of *θ* for various values of Prandtl number Pr.

Fig 6 shows the temperature curves, represented by *θ*(*ζ*), with the change in Prandtl number Pr. Prandtl number gives the ratio of momentum diffusion coefficient to thermal diffusion coefficient. In some manufacturing processes, the Prandtl number can be used to adjust the cooling rate. As emphasized in [53], non-Newtonian fluids have relatively high Prandtl number. At higher Prandtl number, heat convection is dominant over pure conduction. In other words, heat transfer rate at the stretching boundary grows with increasing Pr. The larger heat transfer rate implies shorter penetration depth due to which temperature decreases.

**Fig 7:**
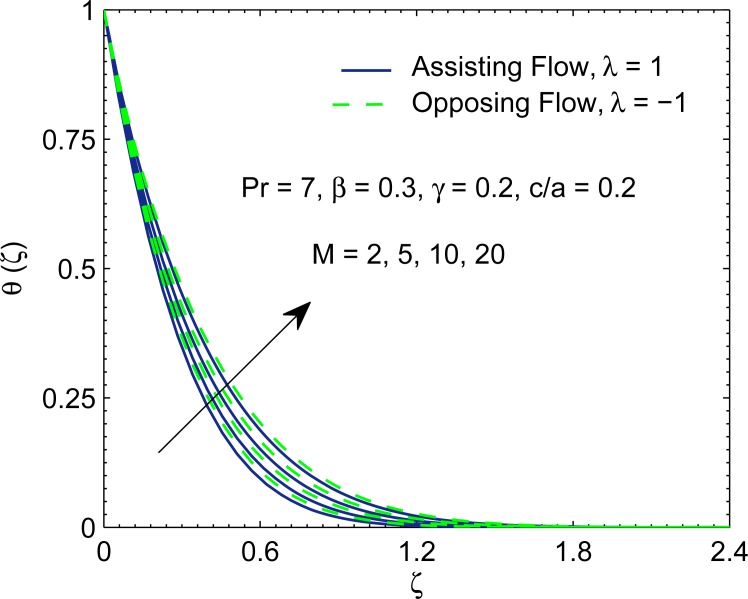
Profiles of *θ* for various values of magnetic interaction parameter *M*.

Temperature curves for varying magnetic interaction parameter *M* are displayed in Fig 7. Fluid temperature in the boundary layer rises for growing magnetic field strength. This is because less hot fluid is carried away from the surface due to reduction in velocity with increasing *M* which in turn yields smaller wall temperature gradient.

**Fig 8:**
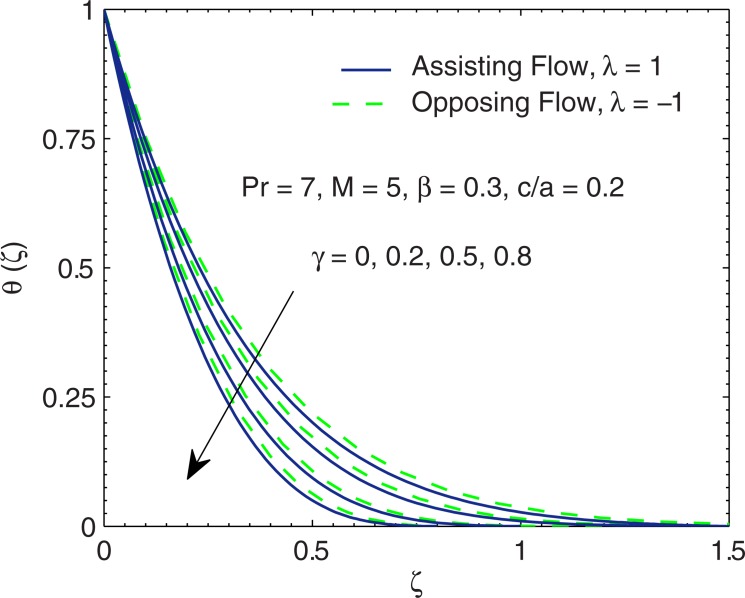
Curves of *θ* for various values of thermal relaxation time *γ*.

In Fig 8, the profiles of temperature *θ* are plotted by varying thermal relaxation time *γ*. We found that temperature *θ*(*ζ*) has a decreasing behavior for increasing thermal relaxation time *γ*. It implies that heat penetration into the fluid reduces as relaxation duration for heat flux increases.

**Fig 9:**
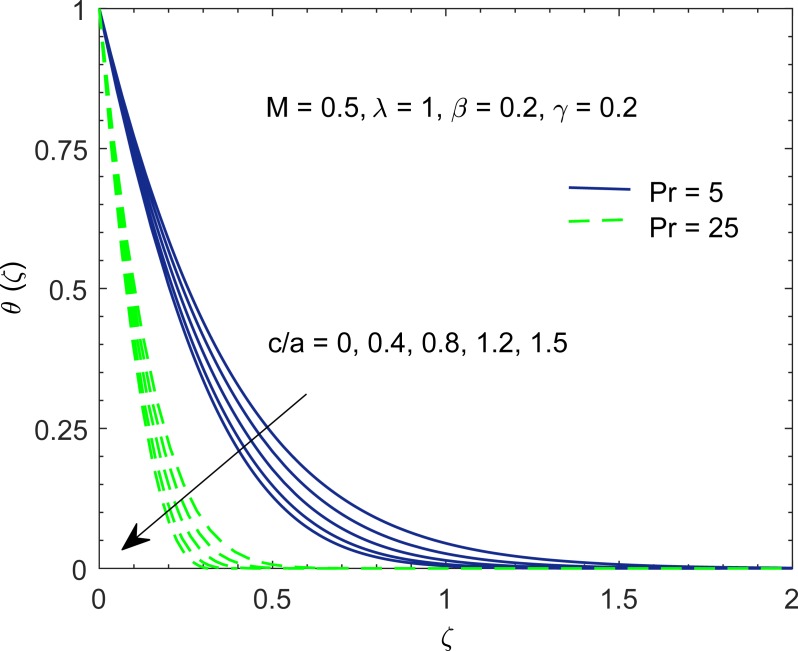
Profiles of *θ* for various values of velocity ratio parameter (*c*/*a*).

The impact of velocity ratio parameter (*c*/*a*) on temperature profile *θ* is depicted through Fig 9. In Fig 3, we observed that fluid flow accelerates in vertical direction with an increment in (*c*/*a*) for *λ* > 0. This eventually intensifies the horizontal flow of cold fluid at the ambient towards hot surface which in turn decreases temperature distribution.

**Fig 10:**
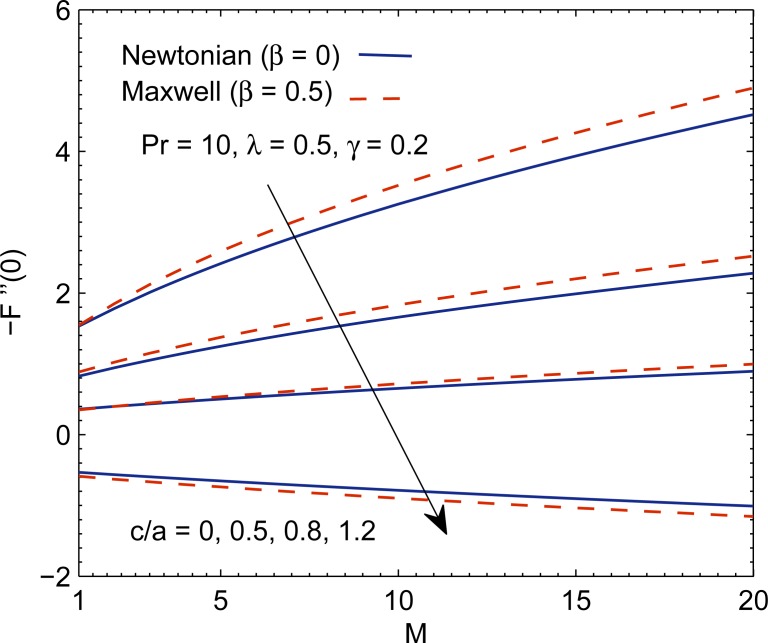
Profiles of −*F*″(0) for various parametric values.

**Fig 11:**
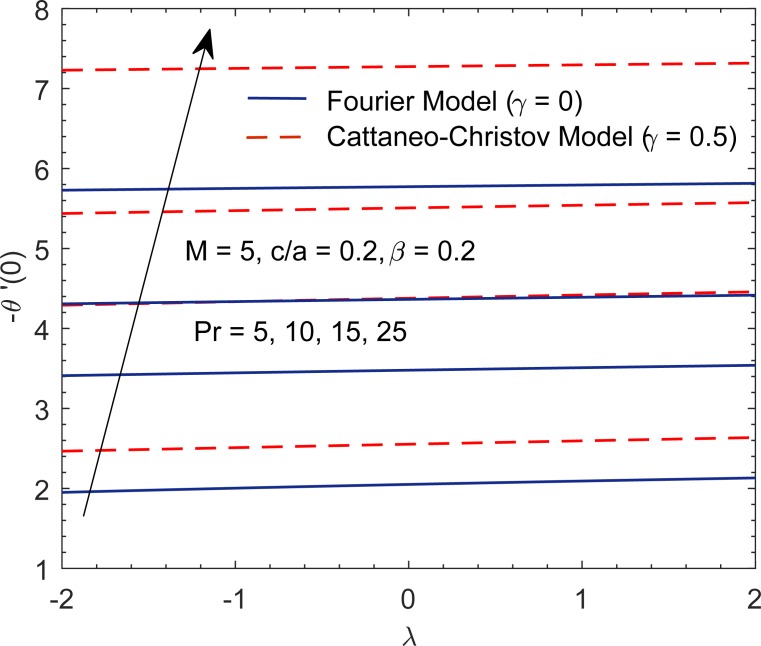
Profiles of −*θ*′(0) for various parametric values.

**Fig 12:**
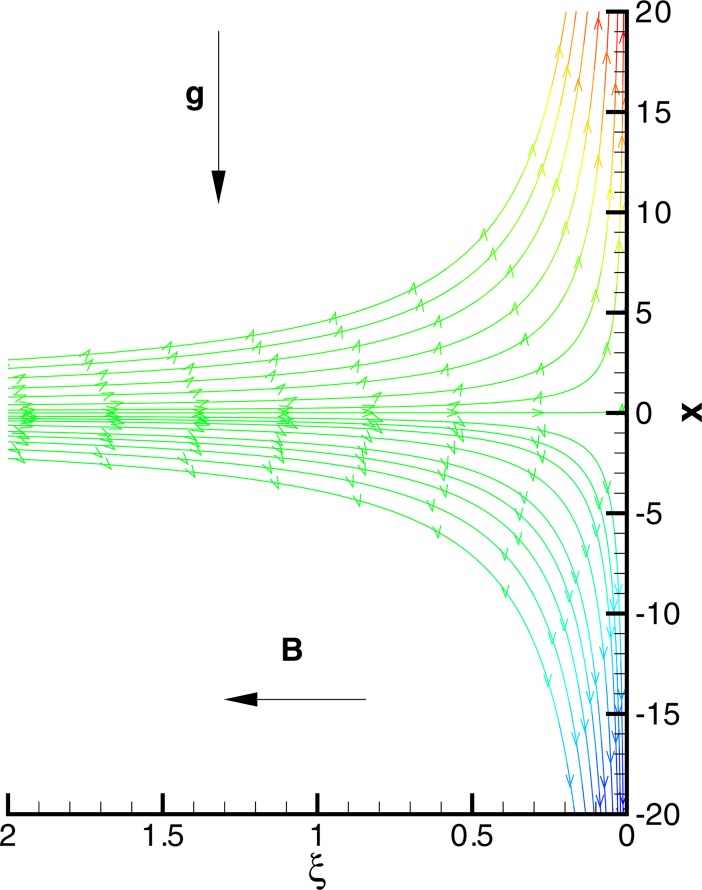
Plots of stream function when *c*/*a* = 0.2, *β* = 0.2, *M* = *λ* = 1.

**Fig 13:**
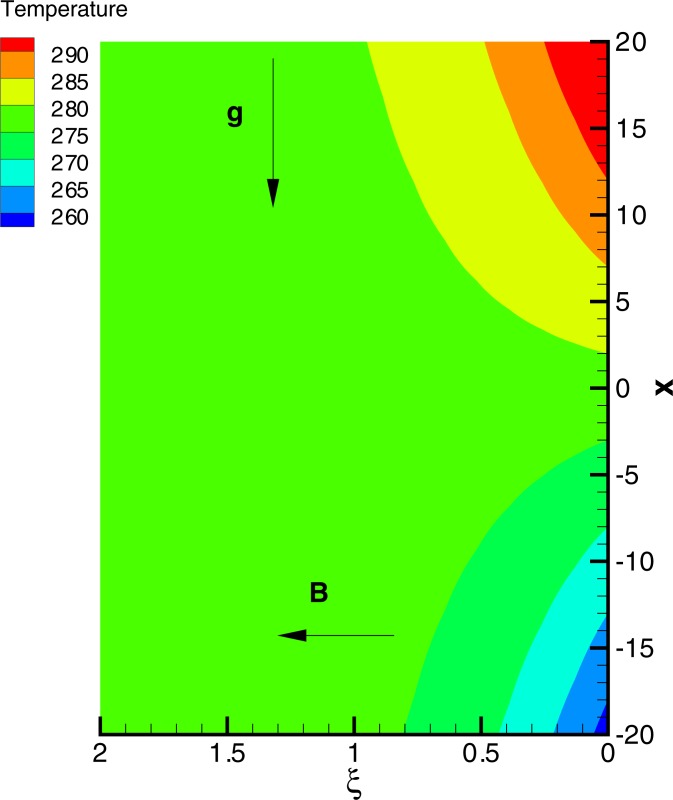
Isotherms for *T*_∞_ = 278*K*, Pr = 10, *γ* = 0.2, *λ* = 1.

**Fig 14:**
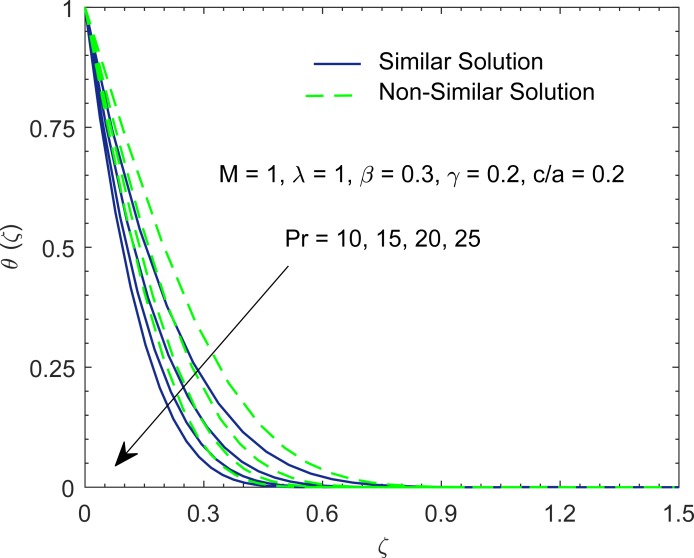
Profiles of *θ* for various values of Prandtl number Pr in both nonsimilar and Similar cases.

Having tested the accuracy of method, we now intend to give physical description to the role of involved parameters on the solution profiles. When (*c*/*a*) = 1, the fluid and stretching boundary have same velocities, which results in no-frictional effect at the fluid-solid interface, that is, *F*″(0) = 0. When (*c*/*a*) > 1, the free stream moves faster than the stretching surface, which implies that fluid applies drag on the boundary due to which *F*″(0) > 0 (see Fig 10). However when (*c*/*a*) < 1, the stretching surface moves faster than the external free stream and hence it applies drag on the fluid. In this case *F*″(0) has a negative sign. In Fig 10, we display the profile of *F*″(0) versus magnetic interaction parameter *M* for a variety of velocity ratio parameters. Magnitude of *F*″(0) continues to enhance as magnetic interaction parameter becomes large. Physically, the reduction in boundary layer thickness due to enhancement in *M* implies an elevation in wall slope of velocity field *F*′. In Fig 11, we give a comparative study of Fourier and Cattaneo-Christov models. Although, qualitatively similar behavior of Prandtl number Pr on *θ*′(0) is found in both models but the variation in *θ*′(0) with increasing Pr becomes prominent as thermal relaxation time become large. Solutions for non-similar partial differential equations have been presented in terms of stream function *ϕ*(*x*,*y*) and temperature *T*(*x*,*y*) in Figs 12 and 13. The profiles of temperature *θ* for constant and variable wall temperature (Similar and Nonsimilar solutions) with variation of Pr are plotted in Fig 14.

## Summary of the results

Cattaneo-Christov heat conduction for mixed convection Maxwell fluid flow adjacent to a heated or cooled vertical surface is investigated here. Flow fields are influenced by vertical magnetic field. Accurate numerical results are presented for broad range of magnetic interaction parameter (0 ≤ *M* ≤ 1000) and moderate values of viscoelastic fluid parameter *β*. Following conclusions are drawn on the basis of current analysis:

○ Transverse magnetic field opposes the momentum transport by deformingsheet while fluid temperature rises for intensifying magnetic field strength.
○ A reduction in momentum boundary layer thickness is anticipated for growing fluid relaxation time.
○ Fluid flow in vertical direction decelerates with increasing magnetic interaction parameter *M* and viscoelastic parameter *β* for (*c*/*a*) < 1. However opposite effect is found in the case where (*c*/*a*) > 1.
○ For any choice of velocity ratio (*c*/*a*), vertical velocity increases/decreases with increasing strength of buoyancy assisting/opposing force.
○ Fluid temperature falls inside the boundary layer as relaxation time for heat flux enlarges.
○ As velocity ratio parameter (*c*/*a*) enlarges, this accelerates the flow of cold fluid at the ambient towards the plate. Consequently, fluid temperature inside the boundary layer falls for increasing velocity ratio parameter (*c*/*a*).
○ Fluid temperature inside the boundary layer increases/decreases as the strength of buoyancy assisting/opposing force increases.
○ Present computations are consistent with those of available articles [34], [36], [52] and [53] in limiting situations.

## Appendix

Here we will present the details concerning the derivation of Eq (2).

Relevant equation governing the two-dimensional flow of incompressible Maxwell fluid along a vertical surface with transverse magnetic field can be expressed as follows:

$$\rho \frac{dV}{dt}=-\nabla p+\nabla \cdot S+\rho g\beta_{T}\left(T-T_{\infty }\right)+J\times B,$$ (17)

in which **V** = [*u*(*x*,*y*),*v*(*x*,*y*),0] denotes the velocity vector, **g** = [0,*g*,0] is gravitational acceleration, **J** = *σ*(**E** + **V** × **B**) denotes the current density in which **B** = [0,*B*_0_,0] is the applied magnetic field and **E** denotes the electric field intensity, *d*/*dt* ≡ ∂/∂t + (**V** ⋅ ∇)**V** is the material time derivative and **S** is the extra stress tensor which obeys the following relation:

$$\left(1+\lambda_{1}\frac{D}{Dt}\right)S=\mu A_{1}.$$ (18)

Here *λ*_1_ is the fluid relaxation time, **A**_**1**_ = (∇**V**) + (∇**V**)^*t*^ is the first Rivlin-Ericksen tensor and *D*/*Dt* the convected time derivative. For any vector **A**, we have:

$$\frac{DA}{Dt}=\frac{\partial A}{\partial t}+\left(V\cdot \nabla \right)A-\left(\nabla V\right)\cdot A,$$ (19)

In order to eliminate **S**, let us assign the operator $\left(1+\lambda_{1}\frac{D}{Dt}\right)$ to Eq (17). We obtain the following:

$$\rho \left(1+\lambda_{1}\frac{D}{Dt}\right)\left[\frac{dV}{dt}-\rho g\beta_{T}\left(T-T_{\infty }\right)-J\times B\right]=-\left(1+\lambda_{1}\frac{D}{Dt}\right)\nabla p+\mu \left(\nabla .A_{1}\right),$$ (20)

Making use of definition (19) and boundary layer approximations, *x*−component of Eq (20) is obtained as follows:

$$\begin{array}{l} uu_{x}+vu_{y}+\lambda_{1}\left(u^{2}u_{xx}+v^{2}u_{yy}+2uvu_{xy}\right)=u_{e}{\left(u_{e}\right)}_{x}+\nu u_{yy}-\frac{\sigma }{\rho }B_{0}^{2}\left(u-u_{e}+\lambda_{1}vu_{y}\right)\\ +g\beta_{T}\left[\left(T-T_{\infty }\right)+\lambda_{1}\left\{uT_{x}+vT_{y}-u_{x}\left(T-T_{\infty }\right)\right\}\right], \end{array}$$ (21)

where we have used ${\left[-\left(1+\lambda_{1}\frac{D}{Dt}\right)\nabla p\right]}_{x}=u_{e}\frac{du_{e}}{dx}+\frac{\sigma }{\rho }B_{0}{}^{2}u_{e}$.
