# Correction: Buoyancy effects in stagnation-point flow of Maxwell fluid utilizing non-Fourier heat flux approach

**DOI:** 10.1371/journal.pone.0200325

**Published:** 2018-07-05

**Authors:** Ammar Mushtaq, Meraj Mustafa, Tasawar Hayat, Ahmed Alsaedi

The third author's name is spelled incorrectly. The correct name is: Tasawar Hayat.

There are errors in Eqs 12, 13, 16 and 20. Please see the correct equations here.

$$\frac{1}{\mathrm{Pr}}\theta^{\prime \prime }+F\theta^{\prime }-F^{\prime }\theta -\gamma \left(F^{2}\theta^{\prime \prime }-FF^{\prime \prime }\theta +{F^{\prime }}^{2}\theta -FF^{\prime }\theta^{\prime }\right)=0\;(PST).$$ (12)

$$\frac{1}{\mathrm{Pr}}\theta^{\prime \prime }+F\theta^{\prime }-\gamma \left(F^{2}\theta^{\prime \prime }+FF^{\prime }\theta^{\prime }\right)=0\;(CWT).$$ (13)

$$\begin{array}{l} \begin{array}{cc} y_{1}^{\prime }=y_{2}; & y_{1}(0)=0\\ y_{2}^{\prime }=y_{3}; & y_{2}(0)=1 \end{array}\\ \begin{array}{cc} y_{3}^{\prime }=\frac{y_{2}{}^{2}-(1+M\beta )y_{1}y_{3}-2\beta y_{1}y_{2}y_{3}-\lambda (y_{4}-\beta y_{1}y_{5})+M(y_{2}-c/a)-{\left(c/a\right)}^{2}}{1-\beta y_{1}{}^{2}}; & y_{3}(0)=s_{1} \end{array}\\ \begin{array}{cc} y_{4}^{\prime }=y_{5}; & y_{4}(0)=1 \end{array}\\ \begin{array}{cc} y_{5}^{\prime }=\frac{\mathrm{Pr}\left\{y_{2}y_{4}-y_{1}y_{5}+\gamma (-y_{1}y_{3}y_{4}+y_{2}{}^{2}y_{4}-y_{1}y_{2}y_{5})\right\}}{1-\mathrm{Pr}\gamma y_{1}{}^{2}}; & y_{5}(0)=s_{2} \end{array}. \end{array}$$ (16)

$$\left(1+\lambda_{1}\frac{D}{Dt}\right)\left[\rho \frac{dV}{dt}-\rho g\beta_{T}\left(T-T_{\infty }\right)-J\times B\right]=-\left(1+\lambda_{1}\frac{D}{Dt}\right)\nabla p+\mu \left(\nabla .A_{1}\right),$$ (20)

There are errors in the last four columns of Table 4. Please see the correct Table 4 here.

**Table 4:** Computational results of −*θ*′(0) for varying values of *γ*,(*c*/*a*) and *β* with *M* = 5.

			Pr = 10		Pr = 25	
*γ*	(*c/a*)	*β*	Assisting Flow *λ* = 1	Opposing Flow *λ* = −1	Assisting Flow *λ* = 1	Opposing Flow *λ* = −1
0.2	0.3	0.2	4.029803 (32.270 sec)	3.967889 (35.197 sec)	6.619606 (60.005 sec)	6.580688 (63.613 sec)
0.4			4.460717 (13.898 sec)	4.399490 (23.047 sec)	7.264644 (36.352 sec)	7.227268 (59.329 sec)
0.6			4.801083 (45.715 sec)	4.773820 (57.069 sec)	7.813688 (13.964 sec)	7.734161 (13.637 sec)
0.8			4.999215 (16.371 sec)	4.962692 (27.048 sec)	7.947736 (8.273 sec)	7.915262 (8.407 sec)
0	0	0.2	3.357325 (2.481 sec)	3.270179 (2.524 sec)	5.659030 (2.871 sec)	5.609332 (2.877 sec)
	0.4		3.641242 (2.737 sec)	3.588133 (2.606 sec)	5.921715 (2.925 sec)	5.883671 (2.921 sec)
	0.6		3.761313 (2.837 sec)	3.715780 (2.651 sec)	6.044662 (2.961 sec)	6.010370 (2.971 sec)
	1.2		4.084024 (2.760 sec)	4.051657 (2.565 sec)	6.395400 (3.526 sec)	6.368817 (2.060 sec)
0.2	0.3	0	4.049712 (38.038 sec)	3.996926 (35.149 sec)	6.638311 (67.316 sec)	6.604845 (60.089 sec)
		0.2	4.029803 (32.270 sec)	3.967889 (35.197 sec)	6.619606 (60.005 sec)	6.580688 (63.613 sec)
		0.4	4.009785 (32.740 sec)	3.939055 (28.188 sec)	6.603513 (67.533 sec)	6.555806 (64.059 sec)
		0.8	3.970128 (27.809 sec)	3.882760 (23.860 sec)	6.566219 (63.526 sec)	6.504412 (61.060 sec)
